# Therapeutic anti-tumor immunity directed against neo-epitopes by intratumor delivery of mRNA encoding MLKL

**DOI:** 10.15698/cst2018.10.160

**Published:** 2018-10-02

**Authors:** Lien Van Hoecke, Xavier Saelens

**Affiliations:** 1 VIB-UGent Center for Medical Biotechnology, VIB, 9000 Ghent, Belgium.; 2 Department of Biomedical Molecular Biology, Ghent University, 9000 Ghent, Belgium.; 3 Department of Biochemistry and Microbiology, Ghent University, 9000 Ghent, Belgium.

**Keywords:** immunogenic cell death, mRNA-treatment, immunotherapy

## Abstract

In recent years, it has become increasingly clear that successful treatment of cancer is possible through the induction of anti-tumor immunity combined with killing of tumor cells. One approach to reach this is to apply cancer vaccines comprising tumor-specific antigens to elicit cellular immunity and chemotherapy to reduce the tumor mass. However, in some cases the dying tumor cell can itself become the vaccine, in particular when the antineoplastic treatment induces so called immunogenic cell death. Immunogenic cell death is characterized by the exposure of damage associated molecular patterns (DAMPs). DAMPs are recognized by innate immune cells which subsequently can prime effector T cell responses against tumor-specific antigens. Unfortunately, many tumors resist exogenous immunogenic cell death stimuli through acquired mutations in cell death signaling pathways. In our recent study (Nat Commun, 9(1):3417), we aimed to overcome these issues through the direct delivery in tumor cells of hypo-inflammatory messenger RNA (mRNA) that codes for mixed lineage kinase domain-like (MLKL) protein, an executioner of necroptosis. This mRNA-based treatment resulted in the potent induction of systemic cellular anti-tumor immune responses that were associated with the regression of the treated as well as distal non-treated tumor cells, as demonstrated in mouse models of transplantable tumors.

We have developed a potentially generic treatment method to induce tumor antigen-specific cellular immunity based on the transient intra-tumor expression of the necroptosis-inducing factor MLKL (**Figure 1**). Importantly, we have focused on the use of *in vitro* transcribed messenger RNA (mRNA) to deliver this cell death inducing factor because (i) mRNA-based gene delivery is very safe, (ii) the technology to produce GMP-compliant mRNA for clinical use is very well established, and (iii) mRNA-based prophylactic and therapeutic vaccination strategies are currently being evaluated in early to late stage-clinical trials for a variety of conditions. The effectiveness of nucleic acid-based therapies crucially depends on a potent delivery system. We opted to deliver the MLKL encoding mRNA by intra-tumor injection followed by electroporation. It involves injection immediately followed by the application of high voltage pulses that transiently disturb membranes and promote electrophoresis of negatively charged nucleic acids. Electroporation has proven to be safe for vaccination and gene therapy with several clinical trials ongoing.

**Figure 1:**
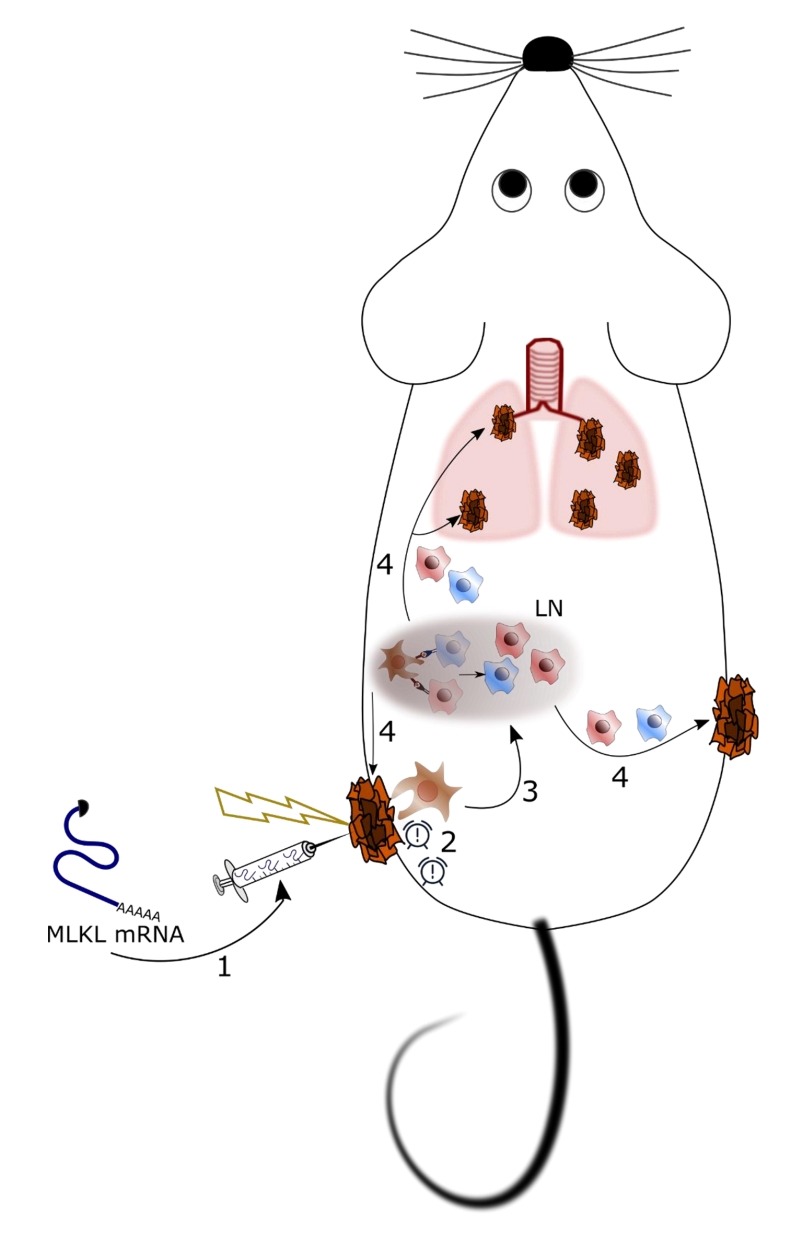
FIGURE 1: Proposed therapeutic mechanism of intratumor treatment with mRNA encoding MLKL. **1.** *In vitro* transcribed hypo-inflammatory mRNA encoding MLKL is injected in an established tumor followed by electroporation. **2.** MLKL-mRNA can enter cells in the tumor mass and evoke cell death with necroptotic hallmarks. Necroptotically dying cells release alarm signals that attract immune cells. **3.** DCs are attracted to the dying tumor cells and will take up tumor-specific antigens and subsequently cross-present these in the draining lymph node (LN) to CD4^+^ and CD8^+^ T cells. **4. **These resulting tumor neo-antigen-specific CD4^+^ and CD8^+^ T cells can than eliminate the treated as well as distal untreated tumors.

mRNA encoding MLKL induced cell death with hallmarks of necroptosis in a variety of tumor cells (**Figure 2**). We next applied mRNA electroporation to tumor cells derived from melanoma and colon carcinoma cell that had been injected subcutaneously in syngeneic mice. *Ex vivo* analysis of the tumor tissue revealed that after MLKL mRNA-treatment, a considerable fraction of the tumor cells had lost their membrane integrity. This treatment resulted in the regression of the treated tumor but, remarkably, also of non-treated tumor cells that were injected in other sides of the mouse.

**Figure 2:**
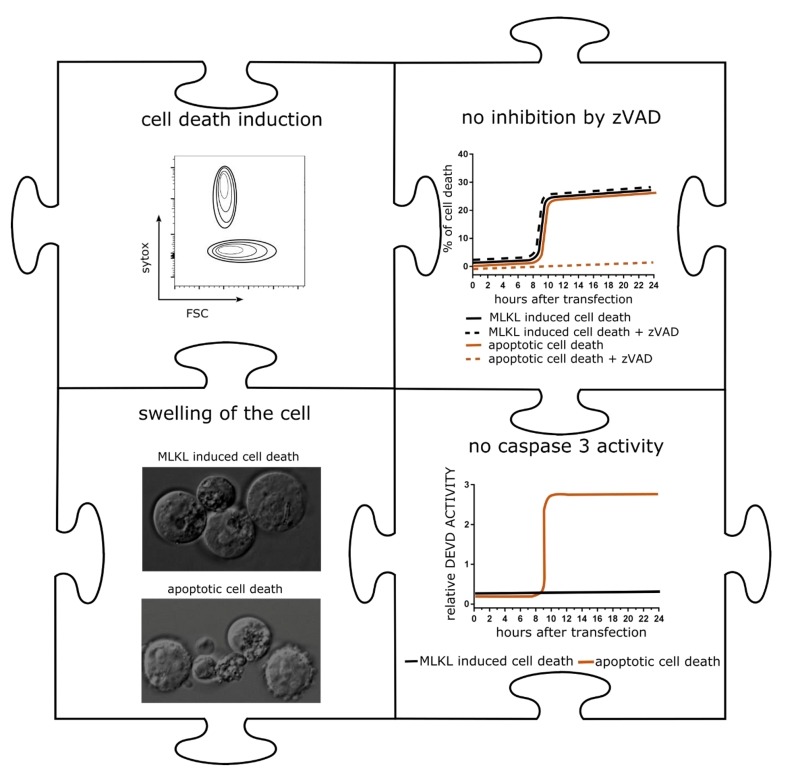
FIGURE 2: Hallmarks of mRNA encoding MLKL induced cell death. Schematic representation of* in vitro *cell death of B16 cells transfected with MLKL-mRNA. **Upper left**: 24 hours after transfection, cells were collected and analyzed by flow cytometry. The graph shows a schematic flow cytometric plot of sytox positive cells. **Upper right:** Impact of the pan-caspase inhibitor zVAD-fmk on cell death induction of apoptotically dying cells or cells transfected with MLKL-mRNA. **Lower right: **Caspase 3 activity over time in apoptotically dying cells or cells transfected with MLKL-mRNA. **Lower left**: Still images of apoptotically dying cells or 24 h after transfection with MLKL-mRNA visualized by time-lapse microscopy.

The standard of care chemotherapy of malignant tumors also induces tumor cell death that may result in an anti-tumor T cell response, which has been well documented for the antracyclin derivative doxorubicin. So what could be the advantage of the MLKL-mRNA treatment over a much simpler drug? To address this, a direct comparison was performed in which the treatment with MLKL encoding mRNA was administered twice intra-tumoral, while doxorubicin was administered every other day. Interestingly, MLKL-mRNA treatment was associated with significantly prolonged survival of the mice compared with the doxorubicin treatment. In addition, the treatment with doxorubicin during three weeks was associated with significantly reduced body weight and lymphocytopenia while the MLKL-mRNA treated group did not display any morbidity.

Nowadays, immune checkpoint inhibitors are the most commonly used type of cancer immunotherapy. This class of drugs works by blocking inhibitory molecules on T cells, antigen-presenting cells, and tumors, thus allowing an enhanced endogenous T-cell-mediated immune response to the cancer. Checkpoint inhibitors were the first class of therapy shown to improve the overall survival for patients with advanced melanoma. Unfortunately, only a fraction of patients benefit from immune checkpoint blockade. In the mouse tumor model, we found that the combination of MLKL-mRNA treatment and systemic administration of a PD-1 inhibiting antibody has a superior antitumor effect compared with either treatment alone. Most likely, the MLKL-mRNA treatment provokes the recruitment of T cells to the tumor bed. Once inside the tumor bed, these T cells might still be silenced by multiple immune suppressive mechanisms used by tumors to evade elimination. Checkpoint inhibitors can take away these breaks.

The mRNA-based treatment rapidly induced CD4^+^ and CD8^+^ T cell responses, which were required to eliminate the tumor. Tumor cells often display epitopes that are derived from so called neo-antigens, which are the products of tumor specific mutations and are ideal targets for cancer immunotherapy. Based on this idea, therapeutic tumor vaccination approaches have been developed that rely on the identification of the tumor specific neo-antigens and the subsequent design of a vaccine that presents these neo-epitopes to the immune system. However, every patient’s tumor possesses a unique set of mutations, known as the mutanome, which must first be identified before a personalized therapeutic vaccine can be applied. This is a time consuming and expensive process that makes the systematic targeting of neo-antigens by vaccine approaches very challenging. We have shown that the MLKL-mRNA treatment can elicit *in situ* immunogenic cell death that results in robust tumor neo-antigen-specific immune responses. This approach primes the adaptive immune system for the induction of neo-antigen specific cellular immunity without prior knowledge of the patient specific mutanome. This way, the tumor, if accessible for MLKL mRNA treatment, effectively becomes its own vaccine.

To take the MLKL-mRNA approach a step closer from bench to bedside, we demonstrated that the new anti-tumor mRNA therapy can also elicit protective anti-tumor responses against a human lymphoma in mice with a reconstituted human adaptive immune system. These findings may open perspectives for the generic clinical development of a MLKL-mRNA-based antitumor treatment. We anticipate that this method would be very safe because mRNA is rapidly degraded. In addition, mRNA production has become economic and scalable and gene expression is fast and transient once the mRNA enters the cytosol. Translating the mRNA injection followed by electroporation to patients is feasible for visible or palpable tumors. In addition, for operable tumor a surgical intervention provides a window of opportunity to inject the mRNA of interest and administer a local electroshock. Formulations of MLKL mRNA that can successfully target tumor tissue after injection, are conceivable future developments.

